# The Case for Selection at *CCR5-*Δ*32*

**DOI:** 10.1371/journal.pbio.0030378

**Published:** 2005-11-01

**Authors:** Pardis C Sabeti, Emily Walsh, Steve F Schaffner, Patrick Varilly, Ben Fry, Holli B Hutcheson, Mike Cullen, Tarjei S Mikkelsen, Jessica Roy, Nick Patterson, Richard Cooper, David Reich, David Altshuler, Stephen O'Brien, Eric S Lander

**Affiliations:** **1** Broad Institute of MIT and Harvard, Cambridge, Massachusetts, United States of America,; **2** Harvard Medical School, Boston, Massachusetts, United States of America,; **3** Laboratory of Genomic Diversity, National Cancer Institute, Frederick, Maryland, United States of America,; **4** Department of Preventive Medicine and Epidemiology, Loyola University Medical School, Maywood, Illinois, United States of America,; **5** Departments of Genetics and Medicine, Harvard Medical School, Boston, Massachusetts, United States of America,; **6** Department of Molecular Biology and Center for Human Genetic Research, Diabetes Unit, Department of Medicine, Massachusetts General Hospital, Boston, Massachusetts, United States of America,; **7** Department of Biology, Massachusetts Institute of Technology, Cambridge, Massachusetts, United States of America,; **8** Whitehead Institute for Biomedical Research, Cambridge, Massachusetts, United States of America,; **9** Department of Systems Biology, Harvard Medical School, Boston, Massachusetts, United States of America; Cornell UniversityUnited States of America

## Abstract

The C-C chemokine receptor 5, 32 base-pair deletion *(CCR5-*Δ*32)* allele confers strong resistance to infection by the AIDS virus HIV. Previous studies have suggested that *CCR5-*Δ*32* arose within the past 1,000 y and rose to its present high frequency (5%–14%) in Europe as a result of strong positive selection, perhaps by such selective agents as the bubonic plague or smallpox during the Middle Ages. This hypothesis was based on several lines of evidence, including the absence of the allele outside of Europe and long-range linkage disequilibrium at the locus. We reevaluated this evidence with the benefit of much denser genetic maps and extensive control data. We find that the pattern of genetic variation at *CCR5-*Δ*32* does not stand out as exceptional relative to other loci across the genome. Moreover using newer genetic maps, we estimated that the *CCR5-*Δ*32* allele is likely to have arisen more than 5,000 y ago. While such results can not rule out the possibility that some selection may have occurred at C-C chemokine receptor 5 *(CCR5),* they imply that the pattern of genetic variation seen at*CCR5-*Δ*32* is consistent with neutral evolution. More broadly, the results have general implications for the design of future studies to detect the signs of positive selection in the human genome.

## Abstract 2

Sabeti and colleagues use dense genetic maps to show that the HIV-resistance *CCR5-*Δ*32* allele is more than 5,000 years old and is likely to have been under mainly neutral selection.

## Introduction

The impact of evolutionary selection on the human population is of central interest and, with increasing information about genetic variation, has become a subject of intense examination [1–6]. Knowledge of selective events and selected loci provide insight into the genetic etiology of human disease, past and present, and into the events that have shaped our species. As infectious diseases pose a major selective force, selected variants may give insight into immunological defense mechanisms—highlighting important pathways in pathogen resistance.

Evolutionary pressure generates a number of potentially detectable signals at a locus under selection as compared to the neutrally evolving genome. Because different populations are subject to distinct selective environments, selection may produce population-specific alleles and greater population differentiation at an affected gene, which can be measured with the*F_ST_* statistic [7]. Positive selection may also cause a rapid rise in an allele's frequency, creating a disparity in the age of an allele estimated from its high frequency in the population (characteristic of an old allele) and its long-range linkage disequilibrium (LD, characteristic of a young allele). LD-based methods such as the Long-Range Haplotype test have been developed to detect this signal [3,8–10].

C-C chemokine receptor 5 *(CCR5)* is one of the most prominent reported cases of recent natural selection in the human genome. First identified as encoding a principal entry receptor for HIV-1 infection of CD4-bearing T lymphocytes, *CCR5* has been the subject of intense focus by geneticists [8,11–14]. A well-established association exists between a 32 base-pair deletion variant in *CCR5 (CCR5-*Δ*32)* and protection from HIV infection, demonstrating that *CCR5* plays an important biological role in HIV entry into cells.

The first suggestion that *CCR5* may have been subject to positive selection was a high proportion of nonsynonymous mutations at *CCR5,* suggesting selective pressure for amino acid divergence [12]. More compelling evidence for selection on *CCR5-Δ32* came from work by Stephens et al. [8]. This study found that *Δ32* occurs at high frequency in European Caucasians (5%–14%, with north-south and east-west clines) but is absent among African, Native American, and East Asian populations, suggesting that the *Δ32* mutation occurred after the separation of the ancestral founders of these populations. Moreover, Stephens et al. [8] reported strong LD between *CCR5-Δ32* and two microsatellite markers, suggesting an estimated age for the allele of only ∼700 y (range 275–1,875 y). The apparent rapid rise in frequency implied strong positive selection, and the specific age raised intriguing possibilities for the selective agent, such as the bubonic plague in Medieval Europe.

With the recent availability of comprehensive information about patterns of allelic diversity in the human genome, we can now reexamine the case for selection at *CCR5* by comparison with extensive empirical data and more sophisticated predicted distributions. We carried out high-density single-nucleotide polymorphism (SNP) genotyping around *CCR5* in multiple populations, and analyzed the data with the benefit of large genomic comparison datasets and revised physical and genetic maps. Our results show that *CCR5-*Δ*32* does *not* clearly stand out in terms of genetic diversity or long-range haplotypes relative to other variants at the locus or throughout the human genome.

## Results/Discussion

We genotyped *CCR5-*Δ*32,* two microsatellites, and 70 SNPs (dbSNP data release 120, www.ncbi.nlm.nih.gov/SNP) extending 837 kbp centromere-distal and 430 kbp centromere-proximal to the *CCR5* locus (Table S1). We studied 340 chromosomes from three populations: European-Americans, Chinese, and Yoruba from Nigeria. Eight of the European-American chromosomes bore the Δ*32* mutation. In addition, we genotyped a subset of the SNPs in 12 Δ*32/*Δ*32* individuals from the original study. This provided a total of 32 chromosomes bearing the Δ*32* allele. We carried out all analyses on both datasets (Table S2).

We first examined the allele frequencies at SNPs around *CCR5* in the European-American, Yoruba, and Chinese population samples for evidence of selection. As a genome-wide empirical comparison, we used two datasets. The first is 2,359 SNPs genotyped in the same 340 samples in the three populations. These SNPs are distributed in 168 immunologic genes from 64 loci across the genome; they were chosen according to the same methodology and have a similar physical distribution as for *CCR5* [15] (see Materials and Methods). The second is data for 63,149 SNPs on Chromosome 3 from the International Haplotype Map Project (HapMap, data release 16) genotyped in the same three populations.

*CCR5* is not a significant outlier relative to the 168 genes or HapMap Chromosome 3 with respect to heterozygosity and *F_ST_* (Table 1; Figure S1). The heterozygosity statistic assesses the genetic diversity in a population; a selective sweep can reduce genetic diversity and balancing selection can increase genetic diversity. The *F_ST_* statistic [7] compares the frequency of an allele between populations; a population-specific selective pressure may produce greater population differentiation at an affected gene. We also looked at the derived allele frequency (DAF) distribution, which can detect the genetic hitchhiking of variation linked to an allele under positive selection, and found no evidence for selection [16] (Table 1; Figure S2). All of these tests have limited power, with genotyping data ascertained to favor common shared SNPs and using the chimpanzee sequence for comparison. Therefore, while the results provide no evidence for selection, it can not be ruled out; this could be further explored with sequencing of a large number of chromosomes.

**Table 1:**
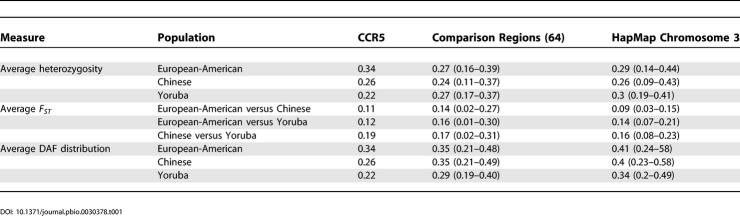
Genetic Diversity at *CCR5* in Comparison with Genetic Diversity for Regions from Two Large Empirical Datasets

We also assessed the significance of the observation that Δ*32* is at moderately high frequency (8%) in the European-Americans but absent in the Chinese and Yoruba populations sampled. The observation is not exceptional in our available polymorphic data: of SNPs present at similar frequency (7%–9%) in European-Americans, ~7% are not found in the Chinese and Yoruba populations for the 168 genes, and 6% are not found for the same populations for the HapMap data. These estimates are likely to be conservative considering that the ascertainment of these studies favors shared polymorphisms. As more data become available, this analysis should be extended by larger sample sizes, more populations, and more closely matched data (including insertion/deletion polymorphisms and functional polymorphisms).

We next tested for signatures of selection by examining the extent of LD around *CCR5-*Δ*32.* For this purpose, we used the Long-Range Haplotype test for selection [3] (see Materials and Methods). Specifically, we calculated the relative extended haplotype homozygosity (REHH), which is sensitive to recent directional positive selection, and extended haplotype homozygosity (EHH), which is more sensitive to multiple selective sweeps at a locus. To estimate the recombination rate, we used two measures: the genetic distance from a family-based linkage study [17] and the number of observed historical recombination events [3] (Material and Methods).

**Figure 1:**
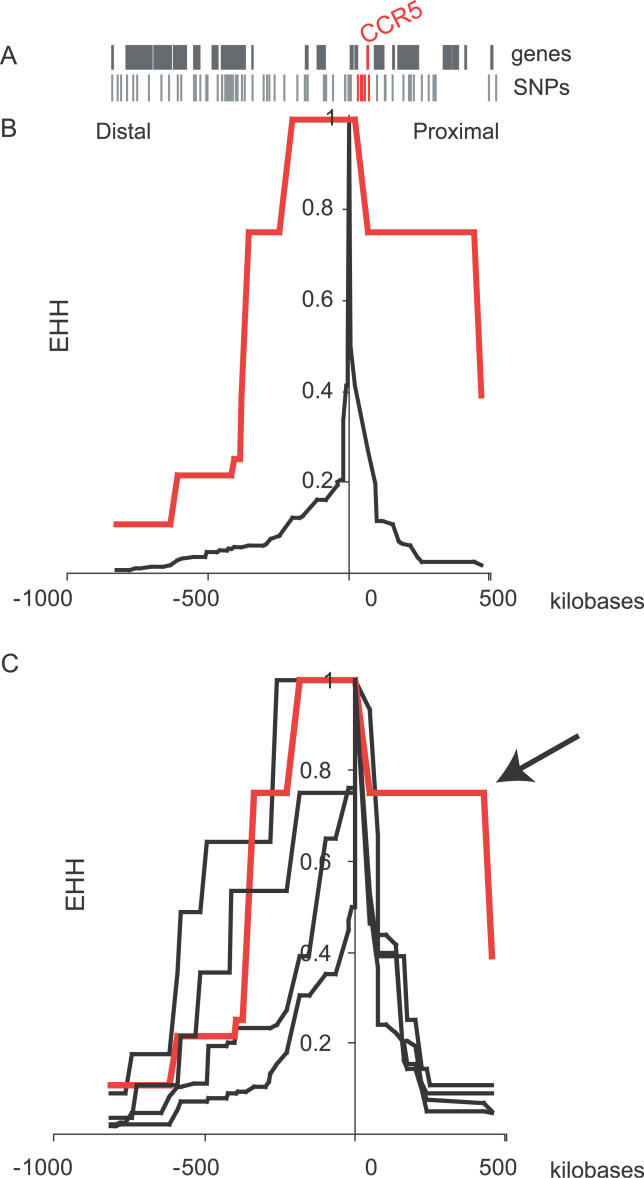
EHH Breakdown of EHH over Distance between the *CCR5-*Δ*32* Mutation and 63 SNPs at Increasing Distances from the Mutation (A) Map of SNPs typed. (B) Comparison between Δ*32* and a single non-Δ*32* class of haplotypes. It should be noted that the Δ*32*-bearing chromosomes appear (red) to have greatly extended LD compared to the non-Δ*32*-bearing chromosomes (black). (C) Breakdown using the eight-marker haplotype containing the Δ*32* mutation. There are five haplotypes in European-Americans (frequencies: 42%, 31%, 10%, 8%, and 8%, respectively). Full haplotype sequences and frequencies in other populations are given in Table S3. Notice that two of the non-Δ*32*-bearing chromosomes (black) appear to have the similar extended LD when compared to the Δ*32*-bearing chromosomes centromere-distal to *CCR5* (red). Centromere-proximal Δ*32*-bearing chromosomes still have the most extended LD, indicated with an arrow.

We initially examined the centromere-distal side of *CCR5* using the approach of Stephens et al. [8] (Figure 1A). Specifically, we sorted the chromosomes into two groups: Δ*32*-bearing and non-Δ*32*-bearing chromosomes. Consistent with the previous study [8], we found that the Δ*32*-bearing chromosomes have much longer LD than non-Δ*32*-bearing chromosomes: the EHH is 5.96 times greater than the average EHH of other variants at this locus (REHH = 5.96 at a distance of 500 kbp or 0.25 centimorgans [cM]) (Figure 1B).

**Figure 2:**
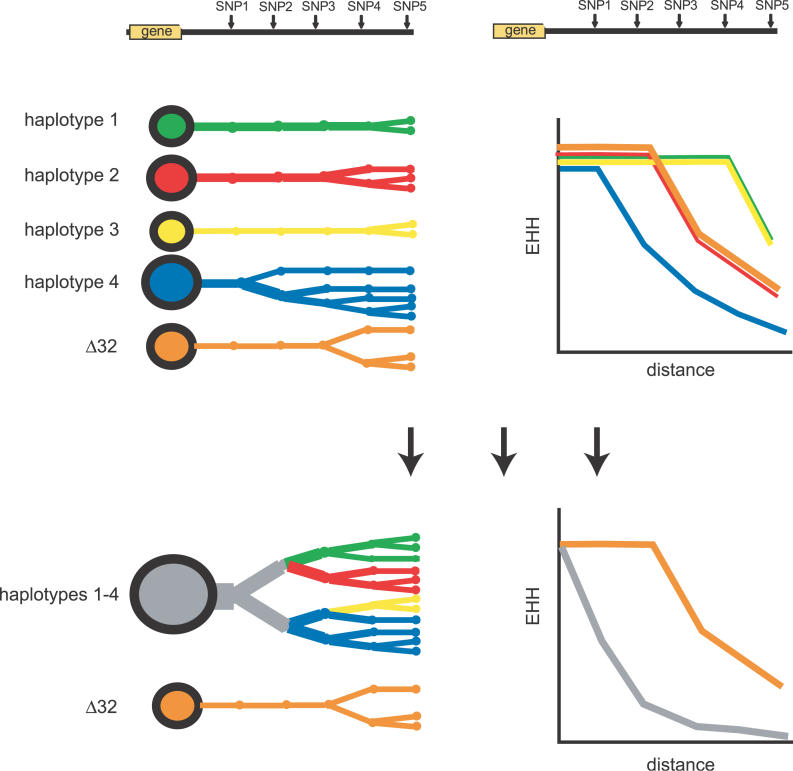
Model of Haplotype-Based Selection Approach The image compares this approach, where the variants at the gene being studied are fully elaborated, to a model where the variants are not fully elaborated. At the top, multiple SNPs are genotyped to fully define the variants that exist in the gene. The resultant observed haplotype structure is shown in both bifurcation diagram and EHH plot formats (see Materials and Methods). At the bottom, only one SNP is genotyped, collapsing all other variants into a seemingly diverse super-haplotype and creating an impression of extension for the remaining haplotype.

We reasoned, however, that the apparent long-range LD might be a result of sorting the chromosome into only two classes based on their genotype at *CCR5-*Δ*32,* rather than dividing them according to the full variation seen at *CCR5.* Figure 2 shows how an apparent signal of long-range LD can readily arise in this fashion. Briefly, one class (for example, the non-Δ*32*) may contain multiple distinct haplotypes whose individual signals of long-range LD may be obscured when grouped together, with the result that the other class (for example, the Δ*32*) appears to have much longer relative LD.

In fact, this is precisely the case for *CCR5.* We fully delineated the variation at *CCR5* by genotyping seven additional SNPs within the gene and defined haplotypes as previously described [18] (Figure S3). There are five distinct haplotypes, including the Δ*32*-bearing haplotype with frequency 8% (Table S3). The relative LD of the Δ*32*-bearing haplotype is significantly lower than for two other haplotypes (REHH = 1.92 versus 6.77 and 3.29 at distance 500 kbp or 0.25 cM; see Figure 1C), indicating that there is no significant evidence of long-range LD on the centromere-distal side of *CCR5.*

We next analyzed LD on the centromere-proximal side of *CCR5.* We first employed the approach used in the original study and again found the Δ*32*-bearing chromosomes had much longer LD than non-Δ*32*-bearing chromosomes (REHH = 20.22 at a distance of 250 kbp or 0.25 cM; see Figure 1B). We then reanalyzed the data by disaggregating the chromosomes into the five haplotypes described above. The relative long-range LD for Δ*32*-bearing chromosomes is much lower (REHH = 7.26), although it is still the highest among the five haplotypes.

**Figure 3:**
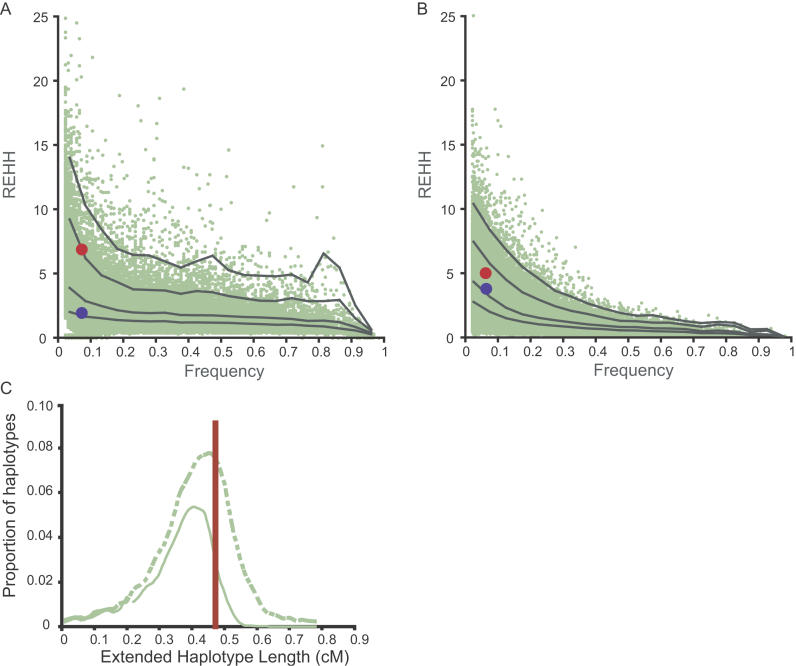
Comparisons with Empirical and Simulation Data (A) and (B) Plots of relative EHH versus frequency for *CCR5* in comparison to HapMap data (release 16) for Chromosome 3 in European-Americans (A) and 1,000 simulations of 400 chromosomes in European-Americans (B). Green dots represent the comparison haplotypes and the lines represent, from bottom to top, the 50th, 75th, 95th, and 99th percentiles. The red dots represent the results for eight *CCR5-*Δ*32*-bearing chromosomes in (A) and 32 *CCR5-*Δ*32*-bearing chromosomes in (B) for the centromere-proximal side, and the blue dots represent results for the centromere-distal side. (C) EHL of the haplotypes of frequency 6%–10% from the HapMap (solid green line) and from simulations (dotted green line) in comparison to *CCR5-*Δ*32* (red line).

We sought to assess whether the extent of LD in the centromere-proximal direction on Δ*32*-bearing chromosomes is unusual relative to that seen across the human genome. We first compared the results to the genome-wide distribution of REHH scores for the HapMap (Release 16, www.hapmap.org, and found that Δ*32*-bearing chromosomes do not clearly stand out from other haplotypes of similar frequency (6%–10%) (Figures 3A and S4). Because the 120 European-American chromosomes genotyped in the HapMap project have limited power for studying low-frequency haplotypes (P. V., B. F., E. S. L., and P. C. S., unpublished data), we augmented the analysis by comparing all 32 Δ*32*-bearing chromosomes to simulations with larger sample size [19] (see Materials and Methods). We simulated 1,000 1-mbp regions in 400 European-American chromosomes under a neutral model, generating 5,915 haplotypes matched with a frequency similar to the Δ*32*-bearing haplotype (6%–10%). The level of EHH for the Δ*32*-bearing haplotype was not unusually high on either the centromere-distal (*p* = 0.49) or centromere-proximal (*p* = 0.15) side of *CCR5* when compared to the level seen at an equivalent recombination distance for the simulated regions. The REHH (we used the EHH of the two common haplotypes for a relative value) was also not unusually high (Figure 3B).

We further examined the extent of the Δ*32*-bearing haplotype in comparison to other haplotypes of similar frequency. For this purpose, we defined the extended haplotype length (EHL) on each side of a haplotype to be the genetic distance at which the EHH score falls to 0.5. The EHL for the Δ*32*-bearing haplotype is 0.212 cM on the centromere-distal side and 0.258 cM on the centromere-proximal side, corresponding to a total of 0.470 cM (Figures 3 and S5). We then determined the EHL for haplotypes of comparable frequency (6%–10%) for both the HapMap data (average EHL is 0.354; *CCR5-*Δ*32* is the 88th percentile) and for the simulated data (average EHL is 0.453; *CCR5-*Δ*32* is the 64th percentile). The distribution is presented in Figure 3. Long-range LD around rare alleles is a prevalent feature in the genome, and the EHL for *CCR5-*Δ*32* therefore does not stand out in comparison to either the HapMap or simulation dataset (Table S4). The EHL for *CCR5-*Δ*32* would only be significant if the recombination rate in this region were several-fold higher than that measured by the current recombinational maps or by counting of historical recombination events (Protocol S1).

Given that long-range LD is a common feature of rare alleles in European-Americans, we wanted to test if our method would have the power to detect selection of an 8% allele over the time scale previously proposed [8]. We simulated 500 regions of 1 mbp length in 400 and 120 European-American chromosomes that had undergone a partial selective sweep beginning either 700 or 2,000 y ago for both groups of chromosomes, carrying the selected allele to a frequency of 8%. We were able to detect recent selection in the 400 chromosomes; 69% of selected alleles originating 700 y ago and 39% of selected alleles originating 2,000 y ago have EHL values above the 95th percentile when compared to the neutral distribution. There is far less power in the 120 chromosomes (30% and 10% of selected alleles originating 700 or 2,000 y ago, respectively), suggesting that the HapMap dataset will be insufficient to scan for rare selected alleles in European-Americans.

Finally, we revisited the estimated date of origin for the *CCR5-*Δ*32* mutation. The original estimate [8] was based on the analysis of two microsatellites that were in strong LD despite apparently being at a considerable genetic distance away (0.91-cM interval and both centromere-distal, according to the genetic maps that were current at the time). With improvements in the genetic map over the past 7 y [17], the microsatellites were shown to be on opposite sides of *CCR5* and at a much shorter genetic distance (0.18 cM, Figure S6). Using the methodology and data employed by Stephens et al. [20] (Table S5), but with the revised genetic map, the estimated age rises from 688 y (275–1,875 y, 95% confidence interval) to 7,000 y (2,900–15,750 y, 95% confidence interval ). When we expanded the analysis to include 32 genetic markers that have been genotyped in the Δ*32*-bearing chromosomes, the estimated age also rises, to a similar value of 5,075 y (3,150–7,800 y, 95% confidence interval). The SNP-based estimate of the age differs and has tighter error bars because the denser map holds more information about historical recombination events than the two microsatellites, whose genetic diversity is roughly equivalent to two SNPs (Figure S7). The older age estimate is consistent with unpublished work on DNA extracted from 3,000-y-old burial sites in central Germany showing that the *CCR5-*Δ*32* was present at an appreciable frequency several millennia ago, at least in central Germany [21].

The revised age estimate suggests the high frequency of the *CCR5-*Δ*32* allele cannot be attributed solely to a strong selective event within the past millennium. If selection did play a role in the high frequency of the allele, the initial selection pressure must have occurred before the period calculated in the previous estimate [8]. It should be noted that the data do not rule out some additional selection occurring within the past millennia, but none that would be detected by the methodology used in Stephens et al. or in the current paper.

Our reanalysis of *CCR5* shows that *CCR5-*Δ*32* does not clearly stand out from the rest of the genome in terms of allele frequency distribution, population differentiation, or long-range LD (Figure S8). The high population differentiation and long-range LD found for *CCR5-*Δ*32* are, in fact, far more common in the genome than previously believed, and therefore do not provide support for the hypothesis of strong selection for *CCR5-*Δ*32.* Using methods described both in the previous study [8] and in the current study, and by examining currently available data, there is no detectable evidence for recent selection for *CCR5-*Δ*32.* Of course, the lack of support does not exclude the possibility of selection for the allele or the locus. Given the biology of the gene, it is certainly possible that it has been subject to some selection despite the lack of clear evidence. We note that small-scale studies of the distribution of mutations [12–14,22] have provided suggestive evidence for selection, but these results may be less convincing in comparison to recently available genome-wide distributions [23].

Beyond the specific results for *CCR5,* our results have important implications for studies of selection in the human genome. First, accurate assessment of LD benefits from fully delineating the core haplotypes at a locus; it may not be sufficient to compare a haplotype of interest to the set of all other haplotypes. Second, long-range LD around specific alleles is a prevalent feature in the genome; the significance of LD results should therefore be assessed relative to empirical distributions observed in genome-wide studies with larger numbers of samples. Third, accurate estimates of an allele's age require accurate genetic maps.

With the growing availability of genome-wide datasets, it should soon be possible to search the genome for signs of strong selective events [3] by studying the pattern of variation at every gene relative to a comprehensive genome-wide distribution. The results should shed light on important factors that have shaped our species and may provide valuable information about natural mechanisms of disease resistance.

## Materials and Methods

### Samples

DNA samples for 93 individuals from 12 multigenerational pedigrees of European-American ancestry were obtained from Coriell Repositories (http://locus.umdnj.edu/ccr). DNA samples from 93 healthy individuals (31 mother–father–child clusters) from the Yoruba in Nigeria were obtained as part of the International Collaborative Study of Hypertension in Blacks. DNA samples from 30 Han Chinese trios from Guanchi were included. DNA samples from a chimpanzee, gorilla, and orangutan were obtained from Coriell Repositories.

### Genotype data

We genotyped 71 SNPs in and around and the *CCR5-*Δ*32* using the mass spectrometry-based MassArray platform provided by Sequenom (San Diego, California, United States), implemented as previously described [18]. The names, locations, alleles, and flanks for all SNPs used are given in Table S1.

Microsatellite genotyping was conducted at the McGill University and Genome Quebec Innovation Center (Quebec, Canada), by use of MultiProbe and MiniTrak Liquid Handling Systems (Perkin-Elmer, Wellesley, California, United States) and 3730 DNA sequencers (Advanced Biosystems, Foster City, California, United States). PCR was performed with fluorescently labeled markers in standard conditions (annealing temperature of 54 °C).

We also used genotypes of 2,359 SNPs, distributed in 168 immunologic genes from 64 loci throughout the genome in the same three populations [15]. SNPs were selected from public databases in multiple batches over a 1.5-y period from July 2002 to December 2003. Preference was given to “double hit” SNPs which have been shown to be more likely to be validated [24]. These criteria may bias our ascertainment of haplotype structure and may reduce the representation of rare and population-specific variation; we comment in the paper where this bias might affect our observations.

We used publicly available data from the International Haplotype Map Project as a comparative distribution of variance in the genome with which to compare our results (http://www.hapmap.org).

### Phasing

We prepared these files using Genehunter (http://www.broad.mit.edu/ftp/distribution/software/genehunter/) to uncover unambiguous phasing using family data [25]. The child chromosomes were then discarded, and we kept only the independent parent chromosomes. We then used PHASE (http://www.stat.washington.edu/stephens/software.html [26,27]) to obtain complete phased data.

### Simulations

We used a computer program that simulates gene history with recombination based on a neutral model of evolution described elsewhere [19,28]. The program was modified to generate data comparable with that collected from the three populations—Chinese, European-American, and Yoruba. The simulations were calibrated to provide data consistent with the HapMap with respect to various genetic measures (including *F_ST_,* heterozygosity, and minor-allele frequency distribution) and used model parameters (including demography and recombination rate) consistent with current estimates [19]. We simulated a long region (1 mbp in length) of DNA and then mimicked the SNP selection strategy used by the SNP Consortium [29], which was the source of most of the SNPs in our study. We modified the program to generate simulations of a partial selective sweep in 400 European-American chromosomes, where 32 chromosomes had a common ancestor 700 y ago as per Stephens et al. [8]. We also tested where the 32 chromosomes had a common ancestor 2,000 y ago.

### *F_ST_*

Mean pairwise distance fixation index*, F_ST_,* was used to calculate genetic differentiation between the three populations [30,31]. *F_ST_* partitions the total variance into within- and between-population components, quantifying the inbreeding effect of population substructure.

### Heterozygosity

Nei's measure of heterozygosity [32], the probability that any two randomly chosen samples from a population are the same, was used to calculate SNP diversity:



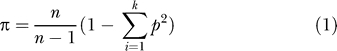



where *n* is the number of chromosomes in the sample, *k* is the number of alleles at a locus, and *p_i_* is the frequency of the *i*th allele.

### DAF distribution

We calculated the DAF distribution for all SNPs where it was likely that the ancestral allele could be determined by genotyping a representative chimpanzee, gorilla, and orangutan. If there was a consensus primate allele across all successfully genotyped primates, it was identified as the ancestral allele. Otherwise, no ancestral allele was defined.

### EHH

EHH assesses the age of each haplotype at a gene by measuring the decay of the extended ancestral haplotype (i.e., SNPs far away from the gene), which occurs over time with recombination. For a population of individuals sharing core haplotype *X,* EHH is the probability that any two randomly chosen samples of core haplotype *X* have the same extended haplotype [3]. It is a measure of the decay of LD across a region of the genome that has two advantages: first, it can be used with multi-allelic markers so a core haplotype model can be studied if desired, and second, it measures LD across a region with many loci and not just between a pair of loci. The EHH is calculated as:



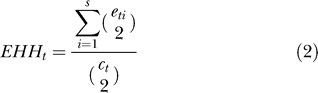



where *t* is the core haplotype tested, *c* is the number of samples of a particular core haplotype, *e* is the number of samples for a particular extended haplotype, and *s* is the number of unique extended haplotypes.

To correct for local variation in recombination rates, we can compare the EHH of a tested core haplotype to that of other core haplotypes present at the locus, using the relative EHH measure (i.e., REHH). REHH is the factor by which EHH decays on the tested core haplotype compared to the decay of EHH on all other core haplotypes combined. One must first calculate the “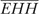 ,” the decay of EHH on all other core haplotypes combined. For this, we use the following equation where *n* is the number of different core haplotypes:



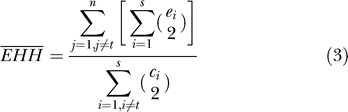



The relative EHH (i.e., REHH) is simply 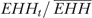 . EHH and REHH were calculated for all haplotypes in all haplotype blocks for *CCR5,* HapMap Release 16 Chromosome 3, and the 1,000 simulated regions (120-chromosome and 500-chromosome sample sets). Haplotypes were placed into 20 bins based on their frequency. *p*-Values were obtained by log-transforming the EHH and REHH in the bins to achieve normality, and calculating the mean and standard deviation. All analysis was carried out using the Sweep software program (P. V., B. F., E. S. L., and P. C. S., unpublished data).

### Observed historical recombination (marker breakdown, all EHH)

When comparing EHH/REHH values across regions, it is important to make sure that the value is being calculated at a similar genetic distance. This will soon be replaced with better cM values, but, where they are not known, this can be matched by the “marker breakdown,” that is the degree to which each added marker at a further distance causes the extended haplotypes to decay for all core haplotypes [3]. This gives an evaluation of how much historical recombination (observed recombinants) has occurred over a distance from the core, and therefore what genetic distance is being looked at. This can be calculated as “all EHH.”



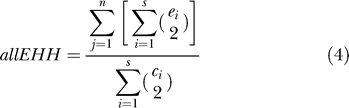



where *n* is the number of different core haplotypes, *c* is the number of samples of a particular core haplotype, *e* is the number of samples of a particular extended haplotype, and *s* is the number of unique extended haplotypes.

### Bifurcation diagram

To visualize the breakdown of LD on core haplotypes, we used bifurcation diagrams [3]. The root of each diagram is a core haplotype, identified by a dark-blue circle. The diagram is bidirectional, portraying both proximal and distal LD. Moving in one direction, each marker is an opportunity for a node; the diagram either divides or not, depending on whether both or only one allele is present. Thus, the breakdown of LD on the core haplotype background is portrayed at progressively longer distances. The thickness of the lines corresponds to the number of samples with the indicated long-distance haplotype.

## Supporting Information

Figure S1 *F_ST_* and Heterozygosity for SNPs within 100 kbp of *CCR5* Compared to 100-kbp Sliding Windows for HapMap Release 16 for European-Americans (54 KB DOC).

Figure S2 The DAF Distribution of *CCR5* Compared to 100-kbp Sliding Windows for HapMap Release 16 for European-Americans (62 KB DOC).

Figure S3 Haplotype Bifurcation Diagrams in European-Americans (231 KB DOC).

Figure S4 The REHH versus Frequency Distribution at Matched Genetic Distance [17] (99 KB DOC).

Figure S5 Estimating the Rate of Degradation of EHH (40 KB DOC).

Figure S6 Remapping of Microsatellite Markers from First Study Given the Improved Genomic Maps (45 KB DOC).

Figure S7 Microsatellite Genotyping (71 KB DOC).

Figure S8 Comparison of Overall Genetic Diversity and Specific Haplotype EHH in Different Populations (38 KB DOC).

Protocol S1 Recombination-Rate Estimates for *CCR5* from Family-Based Linkage Studies (deCODE and Marshfield Maps), from Preliminary Sperm-Typing, and from Population Genetics (LDhat) (32 KB DOC).

Table S1 Information for Δ*32* (rs333), 70 SNPs, and Two Microsatellites Used in the Study (30 KB XLS).

Table S2 *CCR5-*Δ*32* EHH Values for Eight European-American Chromosomes versus the 32 Total Genotyped Chromosomes (23 KB DOC).

Table S3 Haplotype Frequencies for Six Variants in Strong LD at *CCR5,* Genotyped in the Three Population Samples (23 KB DOC).

Table S4 Extended Haplotype Length for Haplotypes of Different Frequency on HapMap Chromosome 3 in European-Americans (23 KB DOC).

Table S5 Details of *CCR5-*Δ*32* Date Estimates (26 KB DOC).

### Accession Number

The LocusLink (http://www.ncbi.nlm.nih.gov/LocusLink) accession number for the C-C chemokine receptor 5 is 1234.

## Abbreviations

cM: centimorgan
DAF: derived allele frequency
EHH: extended haplotype homozygosity
EHL: extended haplotype length
LD: linkage disequilibrium
REHH: relative extended haplotype homozygosity
SNP: single-nucleotide polymorphism
