# Supplementary material for: The Case for Selection at CCR5-Δ32
Source: PLoS Biol. 2005 Nov 1;3(11):e378. doi: 10.1371/journal.pbio.0030378 (PMC1275522; doi:10.1371/journal.pbio.0030378)
Supplement: Supplementary file 1 [file pbio.0030378.sd001.doc]

**Protocol S1: Recombination Rate Estimates**

LD-based tests for selection rely on local recombination rate estimates, which are currently still coarse and variable between sources (Kong et al. 2002; Weber 2002). The estimated recombination rate around *CCR5* is ~0.5cM cM/Mb based on both family-based linkage studies (DEcode and Marshfield maps) and LD studies (LDhat (McVean et al. 2004). For completeness, we also note that a single experiment involving sperm genotyping centromere-distal to *CCR5* yielded an estimated recombination rate of 4.3/Mb, which is ~8 times higher than the estimates from linkage and LD studies.

There is evidence of local hotspots of recombination in the genome, which could be the case here. Alternatively the sperm typing could be an overestimate. Since the experiment involved a two-point measurement of recombination between two nearby markers, it is extremely sensitive to even a low rate of genotyping errors (which are common with sperm typing), and there is no internal measure of this error rate. If the true recombination rate is 0.526 (as indicated by linkage and LD, a genotyping error rate as little as 0.009 would cause the measured recombination rate to be inflated a factor of 8-fold. Such a genotyping error rate would be impossible to detect with the available data and seems quite possible. Because the family studies have a higher genotyping accuracy than sperm typing and have built-in consistency checks (in the form of double-recombinants in multimarker analysis), they are more likely represent the true recombination rate across the region.

It is however an important point that estimates of age and LD based analysis of natural selection are sensitive to measures of recombination rate. We tested if our LD-based assessment of significance at *CCR5* would be sensitive to the larger recombination rate seen in the sperm typing experiments. We found that centromere-distal to *CCR5*, where the sperm-typing was carried out, the REHH and EHH are still not significant with a 4.3cM/Mb estimate (p-values 0.511 and 0.07). The higher recombination rate would change the estimated age from 5550ya to 3460ya.

**Recombination rate measurements from preliminary sperm-typing.** The sperm genotyping was performed using 3 individual donors. We obtained a total of 5209 successful genotypes for 2 microsatellites distal to CCR5 at HG16 positions 46,130,450 (AFMB362wb9) and 46,364,426. We measured a 0.998 cM genetic distance over the 233,875 bp physical distance (4.3cM/Mb).

**Comparison of recombination rate measurements from deCODE, Marshfield, our sperm-typing, and the McVean estimates.** (*) gives the estimate correcting for possible 2 sperms in 1 well (Lazzeroni et al. 1994). For the McVean estimates we used LDhat estimates of recombination rate for the European individuals in the HapMap (HapMap CEU).
