# Supplementary material for: The Case for Selection at CCR5-Δ32
Source: PLoS Biol. 2005 Nov 1;3(11):e378. doi: 10.1371/journal.pbio.0030378 (PMC1275522; doi:10.1371/journal.pbio.0030378)
Supplement: Supplementary file 2 [file pbio.0030378.sg001.doc]

**Figure S1 F_ST_ and heterozygosity for SNPs within 100kb of *CCR5* (red diamond) compared to 100kb sliding windows for HapMap Release 16 for Europeans (gray diamonds, black diamond is average with 95% CI).**
