# Supplementary material for: The Case for Selection at CCR5-Δ32
Source: PLoS Biol. 2005 Nov 1;3(11):e378. doi: 10.1371/journal.pbio.0030378 (PMC1275522; doi:10.1371/journal.pbio.0030378)
Supplement: Supplementary file 3 [file pbio.0030378.sg002.doc]

**Figure S2 The derived allele frequency distribution of *CCR5* (red diamond) compared to 100kb sliding windows for HapMap Release 16 for Europeans (gray diamonds, black diamond is average with 95% CI).** For *CCR5,* 8.3% of SNPs have derived allele frequencies above 80% and 16.7% of SNPs have derived allele frequencies below 5%, which does not stand out from the rest of the genome.
