# Supplementary material for: The Case for Selection at CCR5-Δ32
Source: PLoS Biol. 2005 Nov 1;3(11):e378. doi: 10.1371/journal.pbio.0030378 (PMC1275522; doi:10.1371/journal.pbio.0030378)
Supplement: Supplementary file 4 [file pbio.0030378.sg003.doc]

**Figure S3 Haplotype Bifurcation Diagrams in Europeans** Haplotype variants at *CCR5* observed when we genotype 8 markers in the gene, and breakdown of the long-range LD for each haplotype. By typing only 1 SNP in the gene, the original study collapses the 4 non*-Δ32* variants into a single class, creating an artificial diverse variant.
