# Supplementary material for: The Case for Selection at CCR5-Δ32
Source: PLoS Biol. 2005 Nov 1;3(11):e378. doi: 10.1371/journal.pbio.0030378 (PMC1275522; doi:10.1371/journal.pbio.0030378)
Supplement: Supplementary file 5 [file pbio.0030378.sg004.doc]

**Figure S4 The** **REHH vs. frequency distribution at matched genetic distance.** To assess the significance of the long-range haplotype at *CCR5*, we compared it to the HapMap data (release 16) for chromosome 3 in Europeans using the computer program Finch (Varilly et al unpublished). We examined the full 8 SNP core region tested at 0.25cM distance ((Kong et al. 2002), p-val distal = 0.40, p-val proximal = 0.06). This shows similar results matched historical recombination 0.25cM distance (Figure 3). The grey dots represent data from the HapMap, and the lines represent from bottom to top the 50^th^, 75^th^, 95^th^, and 99^th^ percentiles. The red dots represent the results for *CCR5-Δ32* dataset for the centromere-proximal side and the blue dots represent results for the centromere-distal side. The circles represent results for 8 Δ32 chromosomes and diamonds represent when all 32 ­ chromosomes are included.

i
