# Supplementary material for: The Case for Selection at CCR5-Δ32
Source: PLoS Biol. 2005 Nov 1;3(11):e378. doi: 10.1371/journal.pbio.0030378 (PMC1275522; doi:10.1371/journal.pbio.0030378)
Supplement: Supplementary file 6 [file pbio.0030378.sg005.doc]

**Figure S5 Estimating the rate of degredation of EHH.** We plotted the EHH at increasing genetic distance away from *CCR5-Δ32* in order to estimate the rate of degradation of EHH. There was high correlation between EHH and genetic distance (measured by observed historical recombination distance), giving an associated EHH degradation rate in each direction. Using the degradation rate we could then estimate the extended haplotype length (EHL) the genetic distance at which the EHH degrades to a specified value. We did the same analysis for our comparison datasets from the HapMap and simulations.
