# Supplementary material for: The Case for Selection at CCR5-Δ32
Source: PLoS Biol. 2005 Nov 1;3(11):e378. doi: 10.1371/journal.pbio.0030378 (PMC1275522; doi:10.1371/journal.pbio.0030378)
Supplement: Supplementary file 7 [file pbio.0030378.sg006.doc]

**Figure S6: Remapping of microsatellite markers from first study given the improved genomic maps.** The physical and genetic distances from *CCR5-Δ32* are given for the Stephens et al. paper and the current study. The genetic maps available at the time suggested that the 2 microsatellites were 5 times further away (0.91cM) then proposed by the current genomic and recombination maps (( 339kb and 0.18cM, Supplemental Figure 8) <http://genome.ucsc.edu/> (HG16)(Lander et al. 2001; Kent 2002; Kong et al. 2002)
