# Supplementary material for: The Case for Selection at CCR5-Δ32
Source: PLoS Biol. 2005 Nov 1;3(11):e378. doi: 10.1371/journal.pbio.0030378 (PMC1275522; doi:10.1371/journal.pbio.0030378)
Supplement: Supplementary file 8 [file pbio.0030378.sg007.doc]

**Figure S7 Microsatellite genotyping.** Breakdown of EHH over distance for between the *Δ32* mutation and the 2 microsatellites genoytped in Stephens et al. The microsatellites, AFMB362wb9 and GAAT12D11, have limited genetic diversity equivalent to 2 SNPs (1.83 and 1.40 effective number of alleles, respectively). Map of polymorphisms typed (A). B) Comparison between *Δ32* and all non*-Δ32* haplotypes collapsed into a single class. Notice that the *Δ32*-bearing chromosomes appear (red) to have greatly extended LD compared to the non*-Δ32*-bearing chromosomes (black). C) Breakdown between a 6-marker core containing the *Δ32* mutation. Notice in both cases that 2 of the non*-Δ32*-bearing chromosomes (black) appear to have the similar extended LD when compared to the *Δ32*-bearing chromosomes (red).
