# Supplementary material for: The Case for Selection at CCR5-Δ32
Source: PLoS Biol. 2005 Nov 1;3(11):e378. doi: 10.1371/journal.pbio.0030378 (PMC1275522; doi:10.1371/journal.pbio.0030378)
Supplement: Supplementary file 9 [file pbio.0030378.sg008.doc]

**Figure S8 Comparison of overall genetic diversity and specific haplotype EHH in different populations.** A) The overall long-range diversity for all haplotypes at *CCR5* is surprisingly similar in the 3 populations suggesting the reiterative selection in 1 population did not wipe out old variants and create new variants to make its genealogy younger than other populations. B) and C) EHH for 2 haplotypes shared in the 3 populations. They have remarkably similar values despite different frequency populations suggesting that EHH is dating the origin of the haplotype in an ancestral population and that signal of age was not obscured later due to a specific population history. Note for C, the haplotype is only 7% in Yorubans, so sample-size is likely causing the pattern you see.
