# Supplementary material for: The Case for Selection at CCR5-Δ32
Source: PLoS Biol. 2005 Nov 1;3(11):e378. doi: 10.1371/journal.pbio.0030378 (PMC1275522; doi:10.1371/journal.pbio.0030378)
Supplement: Supplementary file 10 [file pbio.0030378.st002.doc]

**Table S2 *CCR5-Δ32* EHH values for 8 CEPH chromosomes versus the 32 total genotyped chromosomes.** While the EHHs calculated are roughly similar for both sample sizes, there is greater sampling variance in the 8 chromosome dataset. For 8 chromosomes, the 95% confidence intervals is 0.007-0.664 at -550kb and 0.337-0.843 at +261kb. For 32 chromosomes the 95% confidence interval is 0.231-0.626 at -550kb and 0.409-0.840 at +261kb.
