# Supplementary material for: The Case for Selection at CCR5-Δ32
Source: PLoS Biol. 2005 Nov 1;3(11):e378. doi: 10.1371/journal.pbio.0030378 (PMC1275522; doi:10.1371/journal.pbio.0030378)
Supplement: Supplementary file 11 [file pbio.0030378.st003.doc]

**Table S3 Haplotype frequencies for 6 variants in strong LD at *CCR5*, genotyped in the 3 population samples.** F_ST_ is 0.022 between Europeans and Asians, 0.105 between Europeans and Africans, and 0.153 between Asians and Africans. Core homozygosity is 0.29 for Europeans, 0.46 for Asians, and 0.22 for Africans.
