# Supplementary material for: The Case for Selection at CCR5-Δ32
Source: PLoS Biol. 2005 Nov 1;3(11):e378. doi: 10.1371/journal.pbio.0030378 (PMC1275522; doi:10.1371/journal.pbio.0030378)
Supplement: Supplementary file 12 [file pbio.0030378.st004.doc]

**Tables S4 Extended Haplotype Length for haplotypes of different frequency on HapMap Chromosome 3 in Europeans.** We separated the alleles from all haplotype blocks on HapMap Chromosome 3 in Europeans into 20 freqeuncy bins (0-5%, 5-10% ....95-100%). We present here the genetic distance over which these alleles extend, the EHL. We did the same analysis for all haplotype blocks from our 1000 simulations of 400 European chromosomes under a neutral model.
