# Supplementary material for: The Case for Selection at CCR5-Δ32
Source: PLoS Biol. 2005 Nov 1;3(11):e378. doi: 10.1371/journal.pbio.0030378 (PMC1275522; doi:10.1371/journal.pbio.0030378)
Supplement: Supplementary file 13 [file pbio.0030378.st005.doc]

**Table S6 Details of *CCR5Δ32*date estimates**

We estimated the date of origin of *CCR5Δ32* using the methodology and data used in Stephens et al. (Reich and Goldstein 1999) using the revised genetic map. We used two data sets: 1) 146 chromosomes (46 *CCR5Δ32*, 127 non-*Δ32)* genotyped for 2 microsatellies (1 centromere-distal, 1 centromere-proximal) from the Stephens et al. study and 2) 120 chromosomes (32*CCR5Δ32* , 88 non-*Δ*32) genotyped for 39 markers (8 centromere-distal, 29 centromere-proximal).

For each data set, the table presents the best age estimate (and 95% credible interval in parentheses) in generations (age in years were calculated assuming 25 years per generation). The age calculations were done with the ancestral haplotype excluded from the background, and with the ancestral haplotype excluded. The true best estimate must be between these but is probably much closer to the calculation excluding the ancestral haplotype as we have intentionally over-sampled the ancestral haplotype. We also use a likelihood method to combine data from both sides by summing the log-likelihoods from the one-sided analysis. For both the 1-sided and 2-sided data sets, the credible intervals were obtained by using the same likelihood ratio test approach, reporting the full range of generations for which the log-base-10 of the likelihood was within 0.83 of the maximum.
